# Immersive virtual reality for functional hand and finger rehabilitation: results from a randomized controlled trial in 150 patients after traumatic hand injuries

**DOI:** 10.1038/s41746-025-02206-9

**Published:** 2025-12-22

**Authors:** Cosima Prahm, Michael Bressler, Tanja Gohlke, Alexander Hönning, Leila Harhaus-Wähner, Adrien Daigeler, Jonas Kolbenschlag

**Affiliations:** 1 Department of Hand-, Replantation- and Microsurgery, BG Klinikum Unfallkrankenhaus Berlin, Chair of Hand-, Replantation- and Microsurgery, Charité – Universitätsmedizin, Berlin, Germany; 2 Center for Clinical Research, BG Klinikum Unfallkrankenhaus Berlin, Berlin, Germany; 3 Department of Hand, Plastic, Reconstructive and Burn Surgery, Eberhard Karls University Tübingen, BG Klinik Tübingen, Tübingen, Germany

**Keywords:** Health care, Medical research, Neuroscience

## Abstract

Traumatic hand injuries frequently result in prolonged functional impairment. Adherence to conventional rehabilitation is frequently limited by pain, fatigue, and low engagement. Virtual reality systems may increase training volume, but many rely on handheld controllers and lack integration. StableHandVR is an immersive, gamified VR application using optical hand tracking for task-oriented functional hand and finger rehabilitation. In a single-center randomized controlled trial, 150 inpatients undergoing rehabilitation after traumatic hand injuries were assigned to either the StableHandVR intervention (*n* = 75) or an active control (*n* = 75) performing untargeted hand exercises during passive 360° VR exposure. Both groups completed 12 supervised sessions over three weeks. The primary outcome was active range of hand motion (ROM); secondary outcomes included thumb opposition (Kapandji), grip strength, upper-limb function (DASH), pain (NRS), quality of life (SF-36), usability (SUS), intrinsic motivation (IMI), and training adherence. The intervention group achieved significantly greater gains in wrist ROM (+27.8° vs. +17.3°; *p* < 0.001), and thumb opposition (*p* = 0.04). Pain during movement decreased in both groups. Patients using StableHandVR voluntarily exceeded the prescribed training volume by 63%, reporting higher perceived effort (*p* < 0.001), usefulness (*p* = 0.018), and excellent usability (SUS = 85.3). StableHandVR was found to enhance motor recovery, engagement, and adherence, supporting its integration into clinical rehabilitation pathways.

## Introduction

Traumatic injuries to the hand and fingers, such as fractures, tendon lacerations, crush injuries, and amputations, are among the most common causes of occupational disability and functional impairment worldwide. In Germany alone, injuries to the hand account for approximately 40% of all occupational accidents, with an annual incidence of 280,000 cases^1^. Globally, up to 30% of emergency department presentations are due to hand and wrist injuries^2^. These injuries often result in substantial morbidity, prolonged inpatient rehabilitation, delayed return to work, and impaired quality of life.

While surgical reconstruction has advanced considerably, functional recovery remains highly dependent on structured, intensive physiotherapy and occupational therapy. However, adherence to rehabilitation protocols is frequently suboptimal, limited by factors such as pain, fatigue, monotony, and a lack of immediate feedback^3^. A growing shortage of rehabilitation professionals further limits access to adequate therapy for all patients.

In recent years, virtual reality (VR) has emerged as a promising modality to increase engagement, enhance patient motivation, and reduce pain through distraction^4–7^. In stroke rehabilitation, immersive VR systems have been shown to improve upper-limb function, cognitive capacities, and quality of life^8–12^. Scoping reviews focusing on VR for non-neurological upper-extremity conditions reported that VR systems were not inferior to traditional therapy while significantly increasing patient motivation^13,14^. VR, moreover, offers the potential to increase both extrinsic motivation and the objective quality of movement through immersive, playfully designed scenarios and has the potential to establish itself as a conventional treatment modality in orthopedic hand therapy^15^.

Most existing VR rehabilitation tools that target the hand and fingers, however, rely on handheld controllers or touchscreen interfaces, which limit the intuitive use of the hand and restrict fine motor interaction and hinder the full range of motion^16–18^. Moreover, in complex hand injuries, the use of hand-held controllers can be severely limited by the impaired hand function. Optical hand tracking technologies offer a more intuitive and natural form of interaction, enabling the user’s fingers and hand to be directly mapped into the virtual environment^19^. This facilitates fine motor training, enhances embodiment, and potentially increases therapeutic efficacy^20,21^.

Despite these technological advances, most current applications lack clinical depth, biofeedback, or sustained motivational elements^22,23^. Many repurpose standard clinical assessments, such as pegboard tasks or block manipulation, without contextual embedding, relying heavily on the novelty of the VR medium rather than therapeutic structure^24,25^. However, novelty alone does not ensure long-term adherence. For a VR intervention to effectively support motor recovery, it must offer replayability, challenge adaptation, and immersive engagement beyond initial exposure^26^. A high degree of immersion promises to enable a more engaging experience and performance, thus enhancing the positive effects of gamification^27–29^. Tracking the hand and fingers and transferring them into VR enables the user to use them naturally, and will likely create the highest level of immersion for the interaction^30^.

To address these gaps, StableHandVR, a controller-free, hand-tracking-based VR rehabilitation system, was implemented for patients with complex hand trauma. Set in an adaptive farm-themed environment, the system combines conventional rehabilitation exercises with immersive gamified tasks^31^. It provides real-time visual feedback based on individual range of motion (ROM) thresholds, supports repeated use across multiple days, and was developed in collaboration with physiotherapists, occupational therapists, and pain specialists to ensure clinical validity.

The aim of this study was to evaluate the clinical usability and therapeutic impact of StableHandVR during inpatient rehabilitation. Specifically, we investigated whether this system could promote increased voluntary patient activity and support functional improvements during structured rehabilitation. This randomized controlled trial is, to our knowledge, one of the first large-scale evaluations of a fully immersive, controller-free VR rehabilitation system designed specifically for post-traumatic hand and finger injuries.

## Results

**Fig. 1:**
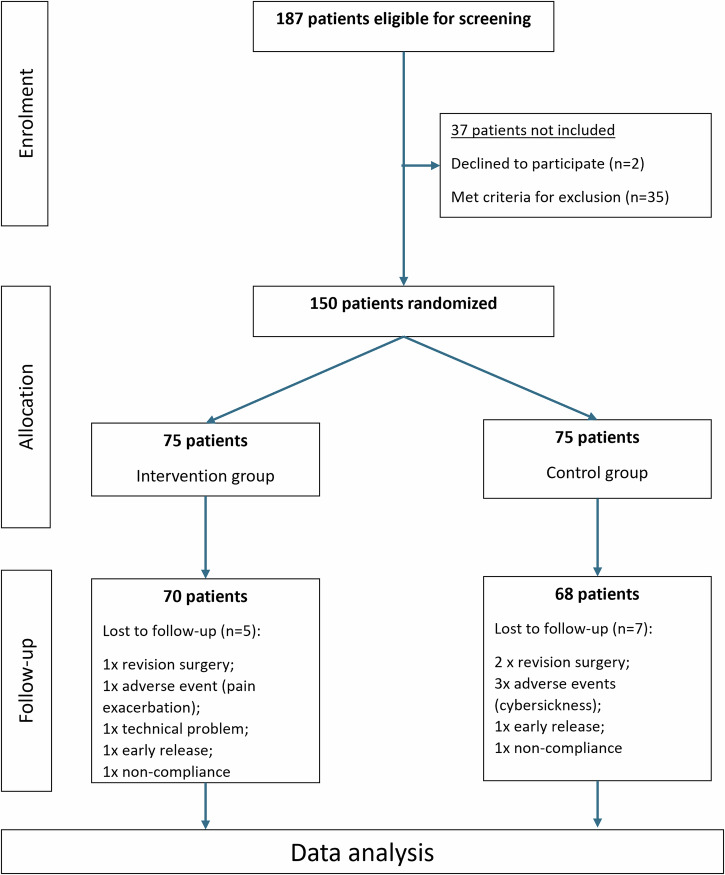
CONSORT flow diagram. A total of 187 patients were screened for eligibility. After excluding 37 patients, 150 patients were randomized into the intervention group (*n* = 75) or control group (*n* = 75). The final analysis included 70 patients in the intervention group and 68 in the control group.

**Fig. 2:**
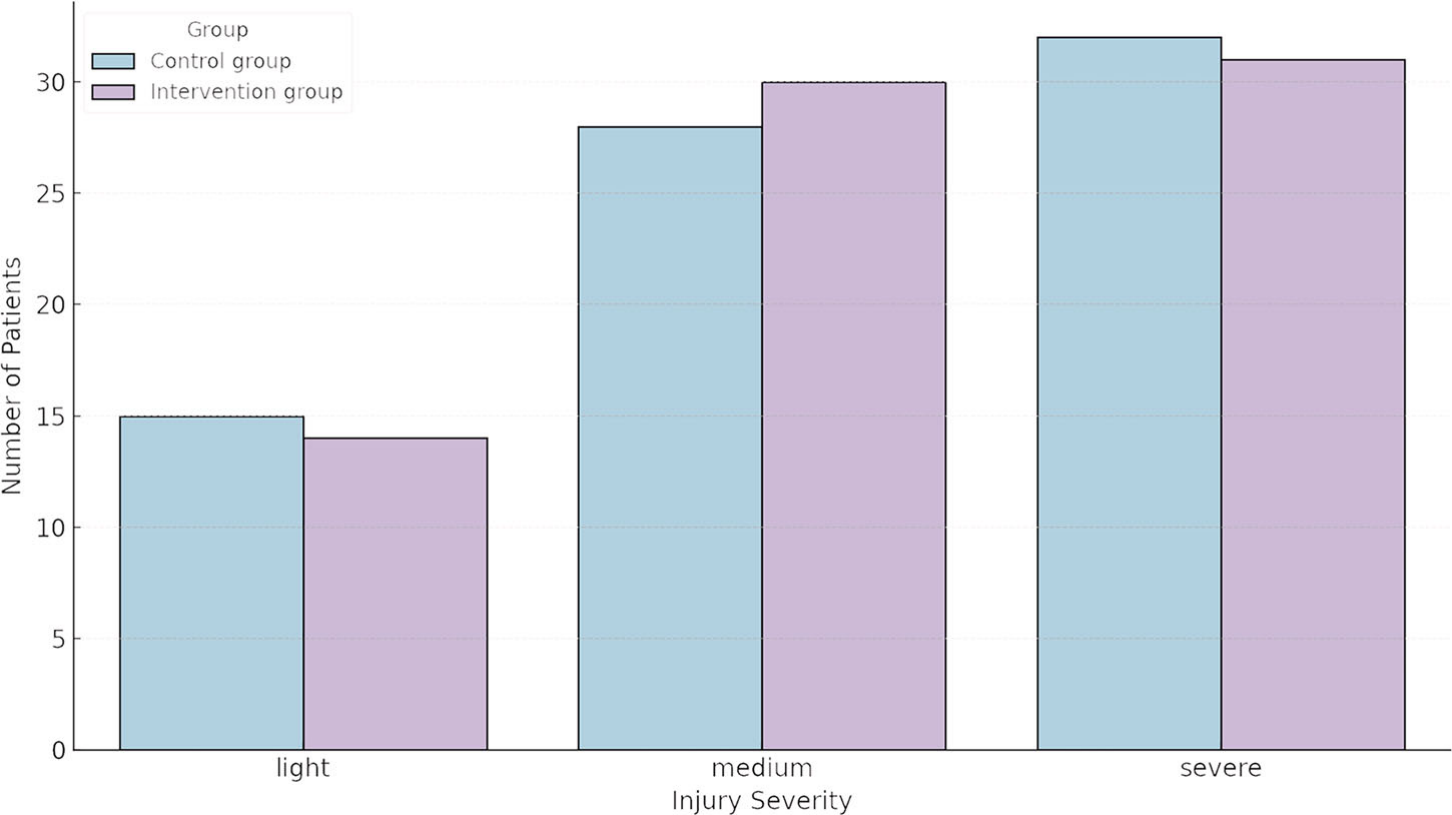
Distribution of injury severity across groups. Bar chart showing the number of patients in each injury severity category (light, medium, severe) for the control and the intervention group. The majority of patients in both groups sustained medium or severe injuries. Randomization resulted in a balanced distribution of injury severity across groups (χ²(2) = 0.229, *p* = 0.892), supporting comparable baseline clinical characteristics between cohorts.

A total of 150 patients with traumatic hand injuries were enrolled and randomized to the StableHandVR intervention group (*n* = 75) or the control group (*n* = 75) (Fig. 1). The mean age was 45.8 years (SD ± 13.8), ranging from 18 to 70 years, with 67% male participants (Table 1). The most common primary diagnoses included metacarpal and phalangeal fractures (ICD-10 S62.5, S62.6), dislocation and strain injuries (S63), flexor tendon injuries (S66), crush injuries (S67), and partial finger amputations (S68). Among all patients, 94.7% (*n* = 142) were right-handed, 4.7% (*n* = 7) left-handed, and one patient (0.7%) reported no clear dominance. The injured side was nearly equally distributed, with 52.0% (*n* = 78) sustaining trauma to the right hand and 48.0% (*n* = 72) to the left. Injury severity was categorized as light, medium, or severe based on clinical assessment of the injured structures. Randomization yielded a balanced distribution of injury severity between groups (χ²(2) = 0.229, *p* = 0.892), confirming comparable baseline clinical characteristics across cohorts. Most patients in both groups presented with medium or severe injuries (Fig. 2). The distribution of physical workload was similar between groups, with 49.3% reporting moderate, 32.7% heavy, and 17.3% light labor intensity prior to injury. In terms of BMI, 42.0% were classified as pre-obese, 33.3% as normal weight, and 22.7% as obese. The median return to work was after 227 days, with a span of 20 to 781 days. Most patients had no prior VR experience (74.7%). No clinically relevant group differences were observed across baseline variables, and all patients had achieved sufficient functional stability to begin active rehabilitation at the start of therapy.

**Table 1:** Baseline characteristics of study participants

Variable	Intervention group (*N* = 75)	Control group (*N* = 75)	All patients (*N* = 150)
Sex, *n* (%)			
Female	29 (38.7)	21 (28.0)	50 (33.3)
Male	46 (61.3)	54 (72.0)	100 (66.7)
BMI, *n* (%)			
Underweight (<18,5)	0	3 (4.0)	3 (2.0)
Normal weight (18.5–24.9)	26 (34.7)	24 (32.0)	50 (33.3)
Pre-obesity(25-29.9)	36 (48.0)	27 (36.0)	63 (42.0)
Obesity(>=30)	13 (17.3)	21 (28.0)	34 (22.7)
Age, Mean ± SD [Range]	44.5 ± 14.4 [18–70]	47.3 ± 13.1 [18–68]	45.9 ± 13.8 [18–70]
Experience with VR, *n* (%)			
Yes	18 (24.0)	20 (26.7)	38 (25.3)
No	57 (76.0)	55 (73.3)	112 (74.7)
Labor severity, *n* (%)			
light	17 (22.7)	9 (12.0)	26 (17.3)
moderate	34 (45.3)	40 (53.3)	74 (49.3)
heavy	24 (32.0)	25 (33.3)	49 (32.7)
missing		1 (1.3)	1 (0.7)
School leaving certificate, *n* (%)			
None	1 (1.3)	2 (2.7)	3 (2.0)
Secondary School Certificate	22 (29.3)	30 (40.0)	52 (34.7)
Intermediate Secondary School Certificate	32 (42.7)	24 (32.0)	56 (37.3)
Technical College Certificate	5 (6.7)	2 (2.7)	7 (4.7)
A-Levels	15 (20.0)	17 (22.7)	32 (21.3)
Affected side, *n* (%)			
Right	37 (50.7)	41 (54.7)	78 (52.0)
Left	38 (40.3)	34 (45.3)	72 (48.0)
Dominant hand, *n* (%)			
Right	71 (94.7)	71 (94.7)	142 (94.7)
Left	3 (4.0)	4 (5.3)	7 (4.7)
None	1 (1.3)	0	1 (0.7)

### Range of motion

In total, 52 active range of motion (AROM) parameters were assessed. ANCOVA revealed significantly greater improvements in 11 ROM parameters in the intervention group compared to controls (*p* < 0.05), including wrist palmar flexion (η² = 0.07), wrist dorsal extension (η² = 0.04), and total wrist AROM (η² = 0.10). Total wrist AROM increased by 27.8° in the intervention group vs. 17.3° in controls (adjusted *p* < 0.001). These improvements correspond to small-to-moderate effect sizes, with the highest in composite wrist motion (Supplementary Table 1 and 2). Notably, in all parameters with significant between-group differences, the intervention group consistently achieved higher gains. Given the large number of individual comparisons (52), the risk of type I errors due to multiple testing cannot be excluded. Accordingly, the reported p-values should be interpreted as exploratory. Nevertheless, in all comparisons with *p*-values < 0.05, the intervention group consistently demonstrated greater improvements than the control group, a pattern that appears unlikely to be attributable to chance alone. No significant interaction effects were found when stratifying outcomes by injury severity, indicating that the intervention was similarly effective across severity levels. Although patients with the most severe injuries showed the greatest numerical improvements, these differences did not reach statistical significance.

### Thumb opposition

**Fig. 3:**
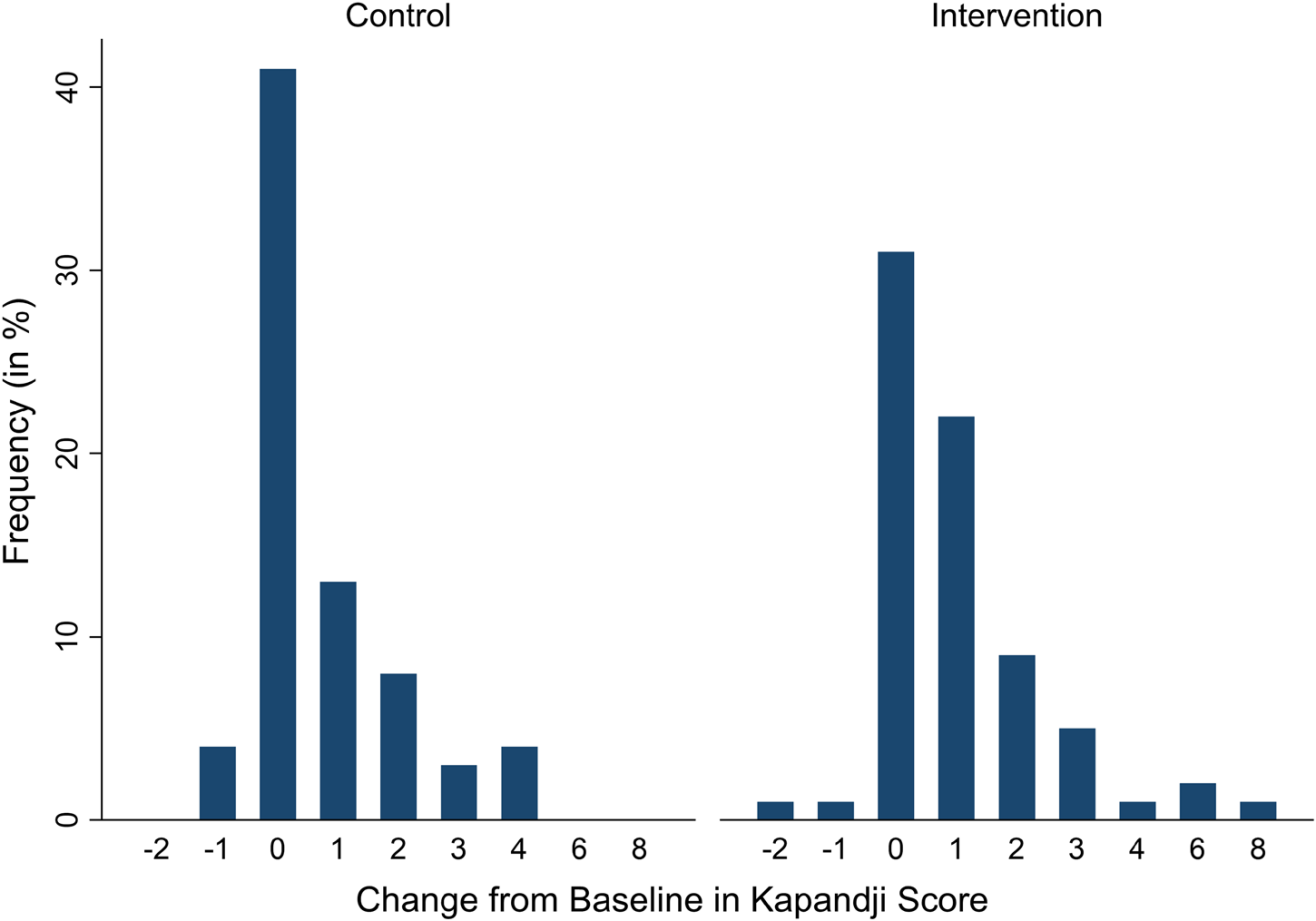
Distribution of change in Kapandji scores from baseline to endline in control and intervention groups. Each bar represents the percentage of participants achieving a given change score. While the control group shows a strong peak at no change (Δ = 0), the intervention group exhibits a broader distribution toward positive outcomes, with more patients achieving improvements of 2 points or greater. This indicates greater gains in thumb opposition following the intervention.

Kapandji Index scores increased in both groups and the within group analysis was significant (*p* < 0.001). The proportion of patients reaching higher Kapandji scores was significantly greater in the intervention group (*p* = 0.04). The odds ratio of 1.69 indicates that patients in the intervention group have a 1.69 times higher chance of achieving a higher Kapandji score at the follow-up measurement time point compared to patients in the control group (Fig. 3).

### Grip strength

Both groups showed significant within-group gains in grip strength (*p* < 0.001) in their injured hand. In the intervention group, maximum grip strength increased by 8.5 kg (right injured hand) and 7.8 kg (left injured hand), while in the control group, increases were 5.0 kg and 6.6 kg, respectively. However, ANCOVA revealed no significant group differences at the endline (all *p* > 0.15; η² < 0.02).

### DASH

**Fig. 4:**
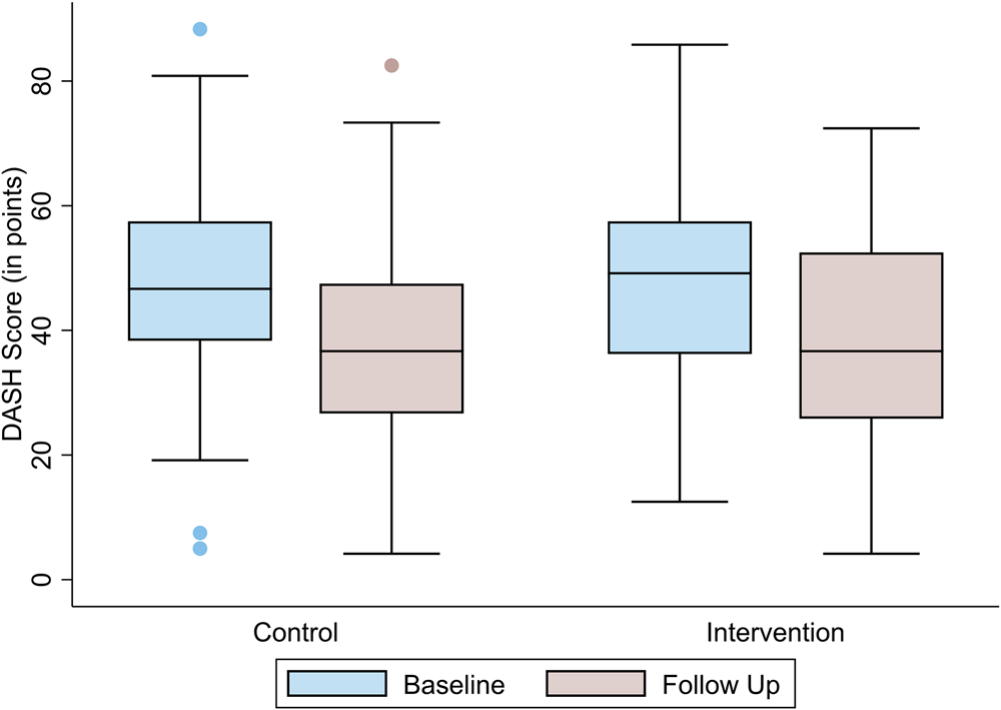
Change in DASH scores from baseline to follow-up in the control and intervention groups. Each box represents the interquartile range (IQR), with the median marked as a horizontal line. Whiskers indicate 1.5 times the IQR, and individual points represent outliers. Both groups demonstrated clinically meaningful reductions in DASH scores over time, with no statistically significant difference between groups at follow-up (*p* = 0.351). A DASH score of zero is considered to have no impairments in daily living.

The DASH score quantifies upper limb disability and symptoms, with lower scores indicating better function. The DASH score improved significantly within both groups (*p* < 0.001 for both). However, no significant group difference was observed at the endline (*p* = 0.4), supporting the non-inferiority of the digital intervention compared to traditional rehabilitation with added passive VR. Both the intervention and control group demonstrated clinically relevant reductions in DASH scores from baseline to follow-up. The mean change from baseline was −9.31 ± 11.82 points in the intervention group and −11.39 ± 13.63 points in the control group (Fig. 4). Despite slightly greater numerical improvements in the control group, analysis of covariance (ANCOVA) revealed no statistically significant difference between groups at follow-up (β = −1.92, *p* = 0.351, η² < 0.01) with medium effect sizes according to Cohen’s d (intervention: d = 0.56; control: d = 0.68).

### Pain

**Fig. 5:**
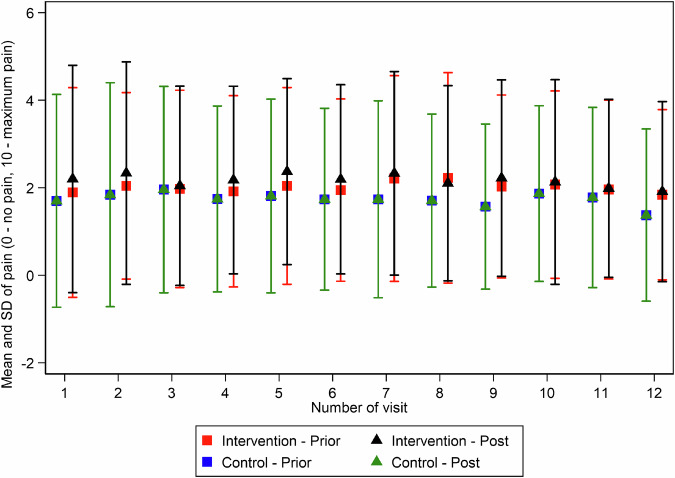
Pain intensity ratings across 12 training sessions for both groups. Mean and standard deviation (SD) of pain ratings before (“Prior”) and after (“Post”) each of the 12 VR training sessions, measured using the 11-point Numeric Rating Scale (NRS; 0 = no pain, 10 = maximum pain). Across sessions, pain levels remained stable, with no consistent intra-session differences and no clear trend toward worsening or improvement. The intervention group reported slightly higher average pain ratings than the control group throughout, although not statistically significant.

Pain intensity was assessed using an 11-point numeric rating scale (NRS), ranging from 0 (no pain) to 10 (worst imaginable pain). In the intervention group, pain at rest remained unchanged from baseline to follow-up (mean ± SD: 1.80 ± 2.22 at baseline and 1.80 ± 2.00 at follow-up), while the control group exhibited a slight reduction (from 1.63 ± 2.30 to 1.25 ± 1.80). Similarly, pain during movement declined in both groups, with a mean change of −0.79 points in the intervention group and −1.16 points in the control group. Ordinal logistic regression adjusted for baseline pain values revealed no significant between-group differences in either pain at rest (β = −0.65, *p* = 0.052) or pain on movement (β = −0.51, *p* = 0.101). Within-group comparisons (Wilcoxon signed-rank test) showed a statistically significant reduction in pain on movement for both the intervention group (*p* = 0.004) and the control group (*p* < 0.001), while changes in pain at rest did not reach statistical significance in either group. Visualizations of pain ratings before and after each individual training session further confirmed a stable pattern over time (Fig. 5), with no meaningful intra-session change and consistently marginally higher reported pain levels in the intervention group.

Notably, the proportion of patients requiring pain medication increased in both groups over the course of the study. However, the increase was more pronounced in the intervention group, rising from 32.2% at baseline to 57.6% at follow-up, compared to an increase from 42.4% to 49.2% in the control group. While reported pain scores showed slight reductions or remained stable, overall medication usage increased, particularly in the VR intervention group.

The majority of patients reported that while using StableHandVR they were “away from their worries”, could “simply switch off” and “immerse themselves” in another world. Attention shifted away from the injury to the game task. The positive influence of visual feedback via color coding (“In the game, I also know from the colors that I’m doing the exercises correctly”) also supported the motivation to move without drawing attention to the pain. In general, the patients noted that they were “more confident because you can’t see your own broken hands” and were therefore able to exercise their hands in larger ranges of motion.

### Quality of Life

SF-36 subscales showed no significant group differences across any physical or emotional domains. Within both groups, 17 of 18 SF-36 subscales improved between baseline and follow-up. The highest gains were achieved in the subscale “role limitations due to physical problems” with more than doubling of the initial values in both the intervention group (mean ± SD: 10.94 ± 22.66 at baseline and 23.83 ± 37.91 at follow-up) and in the control group (mean ± SD: 8.85 ± 22.29 at baseline and 18.85 ± 33.67 at follow-up) followed by the subscales “emotional well-being” in the intervention group (mean ± SD: 61.65 ± 22.97 at baseline and 71.24 ± 21.64 at follow-up) and “role limitations due to emotional problems” in the control group (mean ± SD: 61.62 ± 46.50 at baseline and 70.71 ± 43.96 at follow-up).

### Motivation, adherence, usability, and user experience

**Fig. 6:**
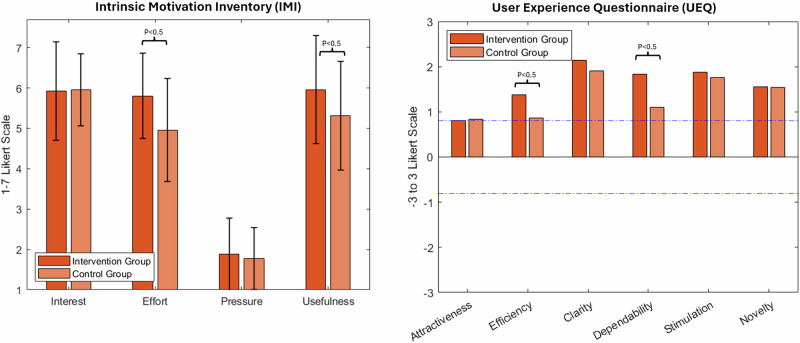
User motivation and game experience ratings for intervention and control groups. (Left) Intrinsic Motivation Inventory (IMI) subscales (Interest, Effort, Pressure, Usefulness) measured on a 7-point Likert scale. The intervention group reported significantly higher scores in “Effort” (p 0.001) and “Usefulness” (p = 0.018). (Right) User Experience Questionnaire (UEQ). Scores range from −3 to +3. Values between −0.8 and +0.8 (marked by blue dashed lines) are interpreted asneutral user experience. Significant differences (p 0.05) between the intervention and control groups were found in two subscales: Efficiency and Dependability).

**Fig. 7:**
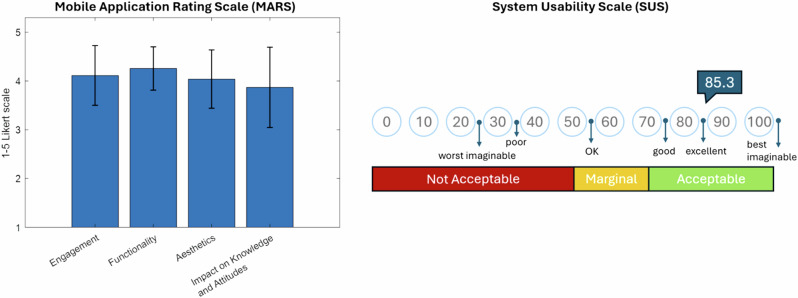
Usability evaluated by the Mobile Application Rating Scale (MARS) and the System Usability Scale (SUS). (Left) Rating of StableHandVR by the patients in the intervention group using the MARS questionnaire on a 5-point Likert scale. With the highest rating on Engagement (mean = 4.12, SD = 0.61) and Functionality (mean=4.26, SD = 0.44). (Right) The StableHandVR system achieved a mean score 85.3 indicating excellent usability on the SUS.

Intrinsic motivation (IMI) scores were significantly higher in the intervention group on the “effort” (*p* < 0.001) and “usefulness” (*p* = 0.018) subscales. System usability (SUS) was rated excellent with a mean score of 85.3. On the User Experience Questionnaire (UEQ), participants rated StableHandVR significantly better in “efficiency” (*p* = 0.003) and “dependability” (*p* < 0.001) domains compared to the VR video control group (Fig. 6). Patients in the intervention group consistently completed more virtual training tasks than the daily minimum prescribed. On average, they interacted with 4.9 (SD 1.3) different task modules per session, exceeding the expected number of three modules. Notably, this was a voluntary behavior without prompting, and remained stable over the 3-week period. Demographic differences in gaming behavior were found in terms of age, gender and previous VR experience, but none of these differences were statistically significant. In terms of patient age, the two youngest age groups played the most stations per day on average and the 40-49 age group played the fewest. In contrast, although the control group sessions were matched in duration to the intervention group (15–20 minutes), adherence to the hand exercise of squeezing a ball while watching 360° VR videos declined significantly over time (*p* < 0.001). For the patients in the intervention group StableHandVR, the quality of the user experience was also assessed using the System Usability Scale (SUS) and the Mobile Application Rating Scale (MARS). The SUS questionnaire resulted in an average value of 85.3, which is in the fourth quartile and stands for excellent user-friendliness (the second highest category). The MARS questionnaire resulted in consistently high values for all four subscales (Fig. 7).

Adverse events were monitored systematically through sessional clinical check-ins and therapist observations using a standardized symptom checklist. Participants were asked to report any discomfort, including pain exacerbation or VR-related side effects. Adverse events were rare: one participant in the intervention group reported transient hand pain during training, while three participants in the control group experienced cybersickness symptoms during passive 360° video exposure^32^.

**Fig. 8:**
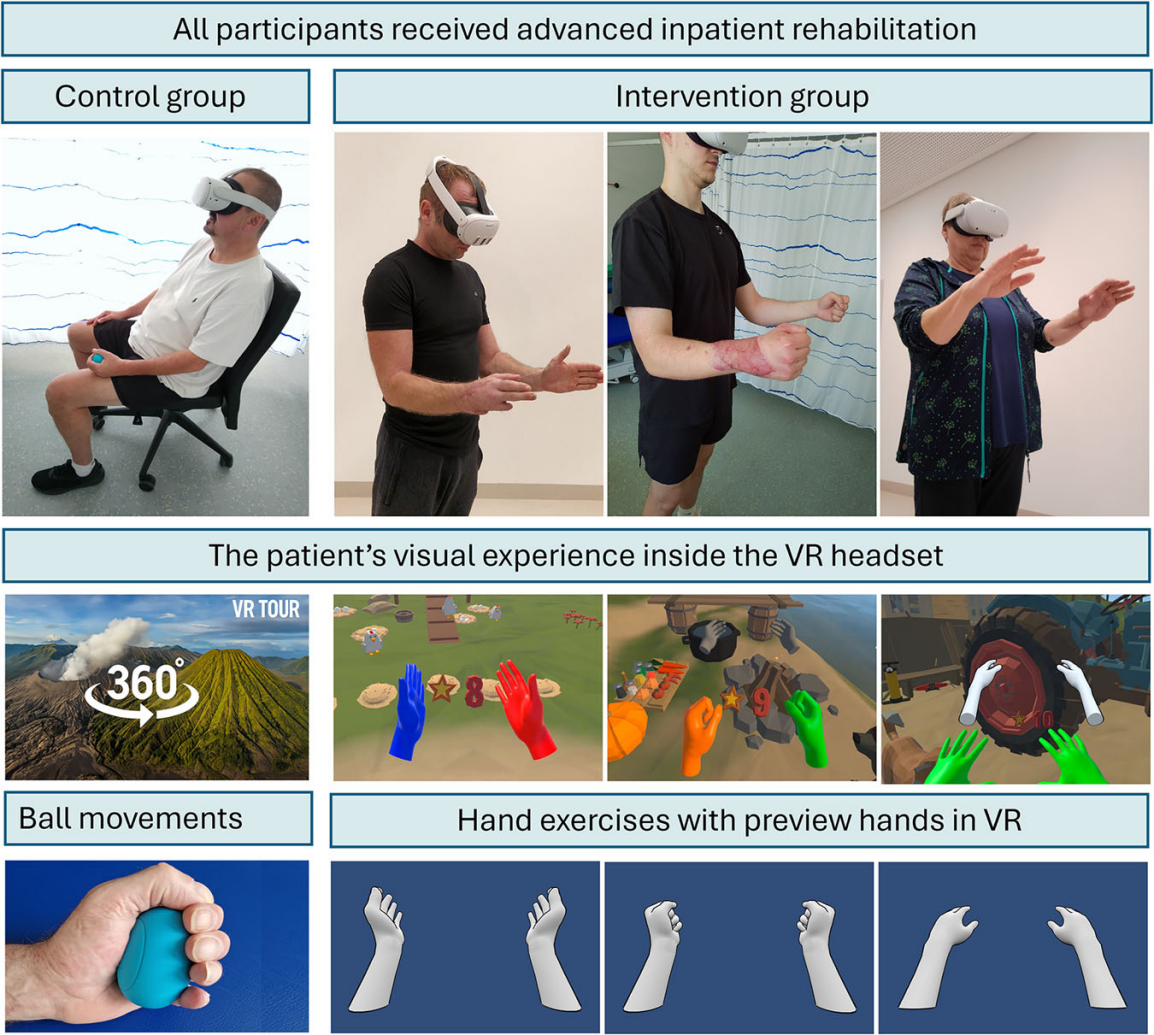
Overview of study interventions and virtual rehabilitation environments. **Top row:** Patients with traumatic hand injuries (e.g., flexor tendon injuries, fractures of the metacarpal and phalangeal bones, amputations) were randomized in either the control or intervention group. **Middle row:** Patients in the control group passively watched 360° VR videos. Patients in the intervention group used StableHandVR, an immersive rehabilitation application set in a farm environment operated via optical hand tracking on the Meta Quest 2 and 3. Traffic light hands provided real-time visual and auditory biofeedback about the movement performance. **Bottom row:** The control group performed repetitive, untargeted, self-paced squeeze–release motions with a soft ball. The intervention group engaged in targeted functional exercises designed to promote specific joint movements (e.g., wrist extension/flexion, thumb opposition), as visualized by the preview hands in grey.

## Discussion

Conventional rehabilitation for hand trauma often faces challenges related to patient adherence, limited engagement, and constrained therapy time. Digital tools such as immersive VR are increasingly investigated to help address these barriers, though few have been evaluated in large-scale, clinically embedded trials. In this context, we conducted one of the largest randomized controlled trials to date investigating the clinical effectiveness of immersive VR in post-traumatic hand and finger rehabilitation. Participants in the intervention group demonstrated greater improvements in active wrist and thumb mobility compared to controls. Self-reported upper limb function (DASH) and health-related quality of life (SF-36) improved similarly in both groups. The intervention group completed more training sessions on average and reported higher ratings for motivation, usability, and system engagement. In total, over 1700 VR sessions were conducted across all participants during the study period. By evaluating StableHandVR under real-world conditions in a complex inpatient rehabilitation setting, this trial provides evidence of both functional benefit and translational potential. The results support the integration of immersive, task-specific VR into structured rehabilitation programs, with particular value in enhancing patient engagement and therapy adherence.

Regarding Motivation, usability, and therapeutic engagement, the high system usability ratings and voluntary overuse observed in the intervention group suggest that StableHandVR successfully addressed key barriers to sustained engagement in hand rehabilitation. The mean SUS score of 85.3 reflects excellent user experience, and patients consistently completed more exercise stations than designated without external prompting. This behavior was paralleled by significantly higher intrinsic motivation scores, particularly in the subdomains of perceived effort and usefulness, indicating that patients were both willing and able to engage intensively with StableHandVR.

The control condition was deliberately designed to isolate the therapeutic contribution of the VR-based, functionally embedded intervention from confounding factors such as novelty, immersion, and increased training dosage. Both groups received the same standardized inpatient rehabilitation (KSR), ensuring a consistent therapeutic baseline. A comparison between StableHandVR and standard therapy alone would have been methodologically weak, as any observed benefit might simply reflect the added training time, rather than the specific efficacy of the digital content. To control for this, we used a matched-duration control involving untargeted hand exercises during passive 360° VR exposure. This approach allowed us to preserve engagement with the VR headset and ensure comparable session lengths, while minimizing the therapeutic specificity of the control. The intention was not to demonstrate that movement per se is effective but to test whether structured, goal-directed VR tasks integrated into meaningful contexts confer additional benefit. Notably, the control condition may have been more engaging than expected, possibly attenuating between-group differences. The StableHandVR system integrates a diverse set of physiotherapy-relevant movement patterns and leverages motivating gamification elements to promote voluntary repetition at functionally effective frequencies. All major joints of the hand and forearm are mobilized toward their physiological limits, thereby improving flexibility and active range of motion. Moreover, the compound movement sequences target coordination and sensorimotor integration, addressing both isolated deficits and complex functional impairments following hand trauma. Its development was grounded in interdisciplinary collaboration with physical, occupational, and pain therapists to ensure both clinical relevance and practical applicability^31^. Following established principles of user-centered design^33^ the system was iteratively refined through close involvement of patients and clinical staff during early prototyping and pilot phases^31^. This participatory, feedback-driven process enabled the early identification of usability issues and facilitated targeted adaptations.

Compared to previous VR rehabilitation approaches, StableHandVR introduces several innovations. Unlike earlier systems relying on handheld controllers or fixed game sequences, StableHandVR uses optical hand tracking coupled with adaptive feedback, enabling real-time performance monitoring without additional hardware. The gamified training structure, featuring multiple contextually embedded farm-based tasks, non-playable characters guiding task progression, and adaptive difficulty, was purposefully designed to increase voluntary engagement and establish a thematic link not only to nature^34^, but also to the occupational context of the inpatient stay. The consistent overachievement of daily training targets in the intervention group reinforces the system’s motivational efficacy and aligns with prior evidence that user motivation is a critical determinant of long-term adherence and rehabilitation success^35^.

These findings support the value of integrating motivational game design principles and intuitive interaction paradigms in digital rehabilitation tools. The ability of StableHandVR to promote autonomous engagement without therapist prompting may not only enhance therapy adherence but also reduce personnel burden in clinical practice.

The analysis of active range of motion (AROM) parameters revealed a consistent pattern of superior gains in the intervention group compared to controls, particularly in wrist mobility and thumb opposition. These differences, while modest in effect size, suggest a specific therapeutic benefit of the StableHandVR system beyond general rehabilitation effects. Thumb opposition, in particular, showed significant improvement and is of high clinical importance given its central role in fine motor tasks, object manipulation, and activities of daily living. These objective improvements in ROM contrast with the absence of significant differences in patient-reported outcomes such as the DASH or SF-36, highlighting a possible dissociation between measurable motor gains and subjective functional perception. This discrepancy may be attributed to the limited sensitivity of self-report instruments to detect fine-grained motor improvements or to the heterogeneity of injury patterns. Furthermore, although current VR systems enable the collection of motion data, their accuracy remains limited in fine motor tasks involving finger articulation^21^. Nonetheless, the consistent superiority of the intervention group across several ROM parameters underscores the value of immersive, repetitive, and goal-oriented training in supporting neuroplastic recovery mechanisms, aligning with prior findings in virtual rehabilitation research. These findings are consistent with earlier studies suggesting that goal-directed VR training enhances motor function by promoting sensorimotor engagement and volitional control^36,37^. Notably, all participants had completed standard outpatient rehabilitation without achieving substantial functional gains prior to their admission to inpatient therapy, where the StableHandVR intervention was implemented. Accordingly, any observed functional improvement can be regarded very highly.

As for the functional self-assessments, both the intervention and control groups exhibited statistically significant improvements in DASH scores over the three-week rehabilitation period, with medium effect sizes observed in within-group analyses. However, no significant between-group differences were detected at follow-up. Notably, the control group also engaged in hand-related activity, specifically, passive exposure to calming nature videos combined with repetitive squeezing exercises, which may have offered a comparable level of sensorimotor stimulation and perceived engagement. Although the study design aimed to minimize novelty effects, qualitative feedback suggests that participants in both groups found their respective interventions engaging. Additionally, the wide range of functional impairments associated with traumatic hand injuries may have reduced the DASH score’s sensitivity to detect group-level differences.

Although the intervention group did not demonstrate superior pain reduction compared to controls, the increased use of analgesic medication in this group likely reflects higher training intensity rather than an adverse effect of the therapy. Participants in the intervention group voluntarily exceeded the prescribed session dose and engaged in more extensive functional movements which, particularly in the early stages of tissue healing, can lead to transient increases in nociceptive signaling. This interpretation is supported by the consistently higher gains in active range of motion and grip strength observed in the intervention group. Notably, patients reported experiencing less focus on pain during VR use, stating they were able to “switch off,” “immerse themselves,” and “forget their injured hand.” One participant noted, “only when you get tired do you realize that you have practiced a lot,” indicating that subjective pain was attenuated during immersive interaction. The discrepancy between subjective distraction and objectively reported pain may point to a dissociation between nociceptive load and cognitive-emotional processing within immersive environments. This aligns with prior evidence that virtual embodiment can effectively buffer pain perception^38,39^. Interestingly, even the generic virtual hand representation provided by the default Meta Quest SDK appeared sufficient to elicit such effects. These findings suggest that well-designed VR systems can sustain high engagement while buffering the psychological impact of pain.

Regarding quality of life, despite functional improvements observed in both groups, no significant between-group differences were found across any SF-36 subdomains. Patients with traumatic hand injuries often face complex biopsychosocial challenges that extend beyond motor recovery, including pain persistence, anxiety regarding return to work, and disrupted identity in relation to manual tasks. These multifactorial influences on perceived quality of life may limit the sensitivity of the SF-36 in detecting subtle but clinically relevant progress during early-stage rehabilitation. It is also important to interpret these findings in the context of the clinical setting. As an occupational accident hospital, many patients may have had pending insurance or pension claims, potentially influencing how they reported their disability and thus may underreport functional improvement in self-assessments such as the DASH or SF-36 questionnaire to safeguard eligibility for long-term compensation. While subjective patient-reported outcomes did not differ significantly, objective functional measures such as ROM and Kapandji confirmed meaningful improvements in both groups. Overall, the DASH and SF-36 may have lacked sensitivity to detect group-level differences in this specific clinical population in the face of social and occupational confounders.

This study has limitations that warrant consideration. While the randomized controlled design enhances internal validity, the monocentric nature of the trial may limit generalizability to other clinical settings, particularly those outside specialized rehabilitation centers. While all patients received advanced rehabilitation in an inpatient setting after having received standard outpatient care, the VR intervention was added on top, meaning we could not restrict participants from receiving guideline-based therapy. Consequently, the additional effect of StableHandVR was evaluated within the context of a high standard of care. This design may have narrowed between-group differences and limited the observable effect size. Furthermore, although the system delivered clinically relevant functional gains, the accuracy of the integrated motion tracking, particularly for fine-grained hand and finger kinematics, remains a technological limitation, even with the advanced capabilities of the Meta Quest 3 headset. Therefore, we measured objective outcomes such as ROM and thumb opposition independently via goniometry. The control group received an active, though untargeted, VR intervention involving 360° video exposure combined with grip-ball exercises. This control condition, while necessary to control for novelty and immersion effects, may have conferred rehabilitative benefit itself, thereby reducing contrast to the targeted intervention. Additionally, in our occupational rehabilitation setting, external factors such as pension concerns may have influenced self-reported measures like the DASH, potentially leading to overreporting of disability. Moreover, hand trauma presents an extremely heterogeneous group of injury patterns. We mitigated this effect by Randomization, selecting patients who underwent an advanced rehabilitation protocol and comparing intra-individual measurements as well.

StableHandVR demonstrated seamless integration into existing therapy routines with minimal training burden for staff. Future research should investigate the long-term effects of home-based use and stratify outcomes across injury subtypes.

## Methods

### Study design

This monocentric randomized controlled trial was conducted with patients undergoing complex inpatient hand rehabilitation. This study investigated whether immersive, gamified VR therapy using StableHandVR could enhance hand function recovery in patients with traumatic injuries and whether this system could promote increased voluntary patient activity. Enrollment took place between January 2023 and March 2025. Randomization was conducted using a Python-based script that generated a 1:1 allocation ratio by assigning random integers (0 or 1). The sequence was uploaded to the REDCap electronic data capture system^40^, which implemented allocation concealment via automated assignment at the point of enrollment. No stratification or blocking was used. A total of 150 patients were randomized to either the intervention group receiving targeted VR therapy or the control group receiving untargeted VR exposure by squeezing an OriOri grip ball (Light&Move Tech) while passively viewing 360° nature videos in VR and listening to a narrator; ball squeezes were automatically logged via an integrated counter (Figure 8). A therapist was present in both groups to supervise headset use, ensure safety, and provide standardized neutral instructions. No individualized coaching or motivational feedback was given during sessions. Compliance was assessed via system log data, which recorded the number of sessions, completed exercises, and their respective durations. Both groups used the Meta Quest 2 and 3 headsets to eliminate novelty or device-related confounders (Fig. 2). The interventions consisted of 12 VR sessions over three weeks, integrated into the standard inpatient rehabilitation schedule. Sessions took place in regular hospital treatment rooms with a 2 × 3 m exercise space.

### Outcome measures

The primary outcome was the functional range of motion (goniometer) and thumb opposition (Kapandji Index). Secondary outcomes included self-reported upper limb function via the Disabilities of the Arm, Shoulder and Hand (DASH) questionnaire, which quantifies physical disability and symptoms relevant to daily activities on a 0-100 scale, where 0 indicates no disability, and 100 indicates the most severe disability^41^. Pain was evaluated via the Numeric Rating Scale (NRS) on a 0-10 measure, analgesic use, and grip strength (dynamometer) were also recorded. Quality of life was assessed with the Short Form Health Survey (SF-36)^42^, a 36-item questionnaire covering eight domains of physical and mental health. Scores range from 0 to 100, with higher values indicating better health status and quality of life. User experience was assessed, with the Intrinsic Motivation Inventory (IMI)^43^, a multidimensional questionnaire that measures perceived competence, interest, and effort during a task. System usability was evaluated with the System Usability Scale (SUS)^44^, a 10-item instrument scored from 0 to 100, where higher scores indicate greater usability and acceptance. User satisfaction and engagement were captured with the User Experience Questionnaire (UEQ)^45^, which evaluates enjoyment, immersion, and esthetic perception during interaction, as well as measuring training adherence. These were selected to reflect clinically relevant domains of functional recovery, patient engagement, and usability. The key hypothesis was that StableHandVR would yield intra-individual improvements comparable to or exceeding those of a control condition involving non-specific VR use. Collectively, these measures aimed to evaluate both clinical efficacy and patient engagement to support reintegration into daily activities. All assessments were performed at baseline and post-intervention.

The study adhered to the Consolidated Standards of Reporting Trials (CONSORT) guidelines. Ethical approval was obtained from the ethics committee of the University of Tübingen, Germany (470/2019B02), and the study was registered under Clinical Trial Registration DRKS00028956 (registered January 5^th^ 2023). All participants provided written informed consent to participate in this study in accordance with the Declaration of Helsinki. Written consent for publication of anonymized data and images was obtained through institutional consent forms.

### Participants

All patients were recruited in a single occupational trauma center during their rehabilitation inpatient stay. Inclusion criteria for all patients were aged 18 or older, occupational traumatic injuries on one or both hands, and normal or corrected-to-normal vision by glasses or contact lenses. All participants were unaware of the experiment’s outcome objectives. Clinicopathologic data, including age, sex, comorbidities, hand pathology, days off work, and adjuvant treatments, were collected from the participants’ electronic medical records. Injury severity was classified as light, medium, or severe based on ICD-10 codes and clinical assessment of functional relevance. Light injuries included soft tissue defects, sprains, dislocations, and stiffness; mediuminjuries included bone fractures, burns, and tendon injuries; severe injuries included nerve damage, amputations, or multi-structure trauma.

### VR Intervention “StableHandVR”

StableHandVR was used on the Meta Quest 2 for the first 6 months and then on the Meta Quest 3 headset when it became available, utilizing its built-in optical hand tracking to allow controller-free interaction^31^. The Meta Quest 3 primarily offered improvements in display quality and wearing comfort, but not in functional hand tracking performance.

The system follows a plug-and-play concept to ensure ease of integration into the clinical setting, requiring no external sensors, cables, or additional hardware, making it suitable for easy use anywhere in the clinic. Optional therapist supervision was possible via tablet streaming, allowing clinicians on the ward to observe patients during training sessions.

The virtual environment was inspired by a 1980s study on postoperative recovery in nature^34^ and thus set on a stylized farm, where patients progress through a series of task-oriented stations, which were provided to the patient by non-playable characters that populate the farm. Each station represents a functional activity, such as feeding chickens, milking cows, cleaning the stable, preparing meals, or repairing a tractor. Every task was subdivided into six movement-based subtasks (e.g., pinch grip, wrist supination, etc.), each repeated ten times, resulting in 60 functional hand exercise repetitions per station. The station’s environment would adapt with each repetition, according to the task (e.g., collecting carrots). The number of stations increased progressively over time, with three new stations unlocked every three days. Players had the option to access additional stations once they completed the day’s exercises, encouraging voluntary continuation of training.

Visual guidance was provided through animated preview hands that demonstrated each target movement in real time. Patients were instructed to mirror these motions using their own hands, which are represented virtually in the scene. Since patients cannot see their physical hands while wearing the headset, these preview hands support motor planning and offer spatial orientation. To ensure clinical relevance, the selection and design of all exercises were guided by a multidisciplinary team of occupational therapists, physiotherapists, and pain specialists, based on established principles of conventional hand therapy^46^. The included movements targeted finger flexion and extension, wrist mobilization, and forearm rotation. In parallel, to support the correct and complete execution of the therapeutic movements and encourage patients to push their individual limits, real-time performance feedback was provided through traffic light hands (Video 1). A visual system that used color coding to reflect movement quality: red indicated the patient had reached their baseline range of motion, yellow signaled moderate extension beyond that baseline, and green confirmed optimal training amplitude. Before each new exercise, the game measured the patient’s initial ROM to establish a personalized baseline. From that point, repetitions were only counted as successful when this threshold was met or exceeded. ROM targets were adapted daily based on the average performance of the preceding sessions. Notably, potential side effects such as cybersickness were actively mitigated during the design of StableHandVR through targeted interaction design and visual stability measures

### Sample Size

The sample size calculation was based on an improvement of the hand function corresponding to a minimal clinically important difference (MCID) in the DASH score. Based on previous studies^47–49^, mean DASH scores after severe hand injuries range from 22 to 50 with standard deviations of around 25. The smallest clinically relevant difference (minimal clinically important difference – MCID) for DASH score is estimated at 10 points^50,51^. Assuming a baseline mean DASH Score of 35 with a standard deviation of 25, 59 patients in each treatment group are necessary to detect a MCID of 10 points in DASH score at follow-up. Assuming a dropout rate of 20%, a sample of *n* = 75 patients per treatment group needs to be randomized. The sample size was calculated using GPower, with a two-sided alpha of 0.05 and a power of 0.85.

### Data analysis

In accordance with CONSORT guidelines, data analyses were pre-specified a priori. Study data were collected and managed using REDCap electronic data capture^40^. Incorrect data entries and measurement errors were identified through programmed plausibility checks and corrected accordingly. As part of a missing data diagnostic, the proportion of missing values for all variables was examined at both assessment time points. Additionally, baseline measurements of non-completers were descriptively compared between treatment groups. Due to low proportions of missing values and an assumed missing completely at random (MCAR) missing data mechanism, no imputation was performed.

### Statistical Tests

Group comparisons of pre-post changes in continuous variables were analyzed using ANCOVA, with the follow-up value as dependent variable, group as factor variable, and baseline value as covariate. If the assumptions for ANCOVA were not met, a generalized linear mixed model was applied. The assumptions were graphically assessed through QQ-plots. Statistical tests to check the normality of residuals, such as the Shapiro-Wilk test, were not carried out, as their results highly depend on sample size. Group comparisons of pre-post changes in ordinal variables were examined using ordinal logistic regression, with the follow-up value as the dependent variable, group as a factor variable, and controlled for the baseline value. Within-group changes in continuous, normally distributed variables were analyzed using a paired t-test. In case of non-normal data and/or ordinal scales, the Wilcoxon signed-rank test was employed. For two-sided tests, a significance level of α = 0.05 was used; for one-sided tests, a significance level of α = 0.025 was applied. All analyses were conducted using STATA (StataCorp LLC, College Station, TX) version 16.1. In addition to statistical significance, the magnitude of the effect was calculated for all comparisons, which provides informationon whether the observed difference is of practical relevance. For group comparisons, the effect size η² was calculated. For pre-post comparisons within groups, Cohen’s d was used for continuous variables, and effect size r for ordinal variables.

## Supplementary information

Supplementary Table 1 + 2 ROM Video 1

Supplementary Video 1

CONSORT_2025_StableHandVR_checklist

## Data Availability

The data recorded and analyzed during the current study are available from the corresponding author on reasonable request up until June 1st, 2035, after which all data will be deleted in line with university data retention policy.
