# Supplementary material for: Immersive virtual reality for functional hand and finger rehabilitation: results from a randomized controlled trial in 150 patients after traumatic hand injuries
Source: NPJ Digit Med. 2025 Dec 22;8:792. doi: 10.1038/s41746-025-02206-9 (PMC12749975; doi:10.1038/s41746-025-02206-9)
Supplement: Supplementary file 1 — Supplementary Table 1 + 2 ROM Video 1 [file 41746_2025_2206_MOESM1_ESM.pdf]

Supplementary Table 1 ROM - Wrist

Variable	n	Mean	SD	Min	Max
Intervention group					
Pronation BL	73	79.66	12.14	20.00	100.00
Pronation FU	73	81.51	7.80	60.00	90.00
Pronation CFB	73	1.85	8.84	-10.00	50.00
Supination BL	73	78.05	20.31	5.00	90.00
Supination FU	73	85.27	11.45	35.00	100.00
Supination CFB	73	7.22	13.38	-10.00	82.00
Radioulnar joints AROM BL	73	157.71	29.90	25.00	190.00
Radioulnar joints AROM FU	73	166.78	17.00	100.00	190.00
Radioulnar joints AROM CFB	73	9.07	18.66	-10.00	92.00
Wrist dorsal extension BL	73	43.49	20.41	-20.00	80.00
Wrist dorsal extension FU	73	54.86	14.41	20.00	80.00
Wrist dorsal extension CFB	73	11.37	11.73	-10.00	50.00
Wrist palmar flexion BL	73	51.44	17.01	10.00	80.00
Wrist palmar flexion FU	73	60.07	12.95	20.00	80.00
Wrist palmar flexion CFB	73	8.63	10.75	-10.00	30.00
Wrist radial abduction BL	73	17.60	8.82	-10.00	35.00
Wrist radial abduction FU	73	20.55	6.10	5.00	35.00
Wrist radial abduction CFB	73	2.95	6.55	-10.00	20.00
Wrist ulnar abduction BL	73	32.60	11.61	0.00	50.00
Wrist ulnar abduction FU	73	37.47	9.86	10.00	50.00
Wrist ulnar abduction CFB	73	4.86	7.68	-10.00	30.00
Wrist AROM BL	73	145.14	48.17	10.00	235.00
Wrist AROM FU	73	172.95	34.52	70.00	230.00
Wrist AROM CFB	73	27.81	22.71	-20.00	80.00
Control group					
Pronation BL	73	80.48	10.81	20.00	90.00
Pronation FU	73	82.60	6.88	60.00	90.00
Pronation CFB	73	2.12	10.20	-10.00	70.00
Supination BL	73	73.97	26.98	-50.00	90.00
Supination FU	73	79.04	23.21	-50.00	90.00
Supination CFB	73	5.07	13.37	-20.00	70.00
Radioulnar joints AROM BL	73	154.45	32.71	40.00	180.00
Radioulnar joints AROM FU	73	161.64	25.07	40.00	180.00
Radioulnar joints AROM CFB	73	7.19	21.73	-20.00	140.00
Wrist dorsal extension BL	73	44.25	21.61	-40.00	80.00
Wrist dorsal extension FU	73	51.71	17.14	0.00	80.00
Wrist dorsal extension CFB	73	7.47	11.52	-20.00	60.00
Wrist palmar flexion BL	73	47.74	24.08	-20.00	90.00
Wrist palmar flexion FU	73	52.53	21.35	-30.00	90.00
Wrist palmar flexion CFB	73	4.79	10.85	-30.00	40.00
Wrist radial abduction BL	73	15.82	8.98	-5.00	30.00
Wrist radial abduction FU	73	17.74	8.12	0.00	40.00
Wrist radial abduction CFB	73	1.92	5.31	-10.00	15.00
Wrist ulnar abduction BL	73	30.96	14.43	-30.00	55.00
Wrist ulnar abduction FU	73	34.04	12.15	0.00	55.00
Wrist ulnar abduction CFB	73	3.08	6.44	-10.00	30.00

Wrist AROM BL	73	138.77	56.59	0.00	235.00
Wrist AROM FU	73	156.03	48.88	0.00	235.00
Wrist AROM CFB	73	17.26	23.38	-40.00	120.00

BL – Baseline, FU – Follow Up, CFB – Change From Baseline

Supplementary Table 2 ROM – Significant between-group differences resulting from ANCOVA

Variable at FU, adjusted for BL	R^2	β	t	p-value	η^2
AROM wrist	0.825	-12.28	-4.08	<0.001	0.10
Wrist palmar flexion	0.744	-4.85	-3.18	0.001	0.07
Digitus Manus III PIP flexion	0.783	-3.90	-2.69	0.004	0.05
Wrist dorsal extension	0.704	-3.63	-2.52	0.007	0.04
Wrist ulnar abduction	0.714	-2.25	-2.26	0.013	0.03
Wrist radial abduction	0.571	-1.74	-2.17	0.016	0.03
Supination	0.699	-3.63	-2.14	0.017	0.03
Digitus Manus V PIP extension	0.788	-2.37	-1.79	0.038	0.02
Thumb opposition	0.773	0.26	1.78	0.038	0.02
Digitus Manus I AROM ext flex	0.736	-5.36	-1.81	0.037	0.02
Digitus Manus I radial abduction	0.643	-1.88	-1.82	0.035	0.02

BL – Baseline, FU – Follow Up, R^2 - Coefficient of determination, β – Regression coefficient, t – t value, η^2 - Effect size

Supplementary Video 1

Guided Hand Movements with real-time Visual Feedback in StableHandVR*. These videos show therapeutic hand and finger exercises that are embedded in an interactive farm-themed environment, where users engage in playful tasks such as milking cows or repairing machinery to train range of motion and dexterity. Visual cues, including preview hands and traffic light hands, guide correct execution and support motor learning. In the PDF version of this article, please click anywhere on the figure or caption to play the video in a separate window.*
